# Racial differences in long-term social, physical, and psychological health among adolescent and young adult cancer survivors

**DOI:** 10.1186/s12916-023-03005-3

**Published:** 2023-08-04

**Authors:** Sooyeon Kim, Juhee Cho, Dong Wook Shin, Su-Min Jeong, Danbee Kang

**Affiliations:** 1 Department of Clinical Research Design and Evaluation, Samsung Advanced Institute for Health Science and Technology, Sungkyunkwan University School of Medicine, 115 Irwon-Ro, Gangnam-Gu, Seoul, 06355 Republic of Korea; 2 Center for Clinical Epidemiology, Samsung Medical Center, Sungkyunkwan University School of Medicine, Seoul, Republic of Korea; 3 Department of Family Medicine, Samsung Medical Center, Sungkyunkwan University School of Medicine, Seoul, Republic of Korea; 4 Department of Digital Health, SAISHT, Sungkyunkwan University, Seoul, Republic of Korea; 5 Department of Medicine, Seoul National University College of Medicine, Seoul, Republic of Korea

**Keywords:** Adolescent and young adult, Cancer survivor, Long-term effect, Psychosocial, Survivorship

## Abstract

**Background:**

The current guidelines for survivorship in adolescents and young adults (AYA) cancer are based on studies conducted in the United States and European AYA survivors. However, previous studies have shown that the health-related quality of life in cancer survivors can vary depending on race, yet the long-term health differences among AYA survivors by race/ethnicity have not been fully explored. Therefore, our aim is to compare the psychosocial and physical health of AYA survivors and their matched controls across different racial and ethnic groups.

**Methods:**

We conducted a cross-sectional study using US National Health and Nutrition Examination Survey (NHANES) and the Korea NHANES from 2007 to 2018. We included AYA cancer survivors who were diagnosed with any type of cancer aged between 15 and 39 years, and who were adult with aged over 18 years old at survey year. We then stratified the study population by race/ethnicity with Non-Hispanic White (NHW, *n* = 310), African American (AA, *n* = 42), Hispanic (*n* = 81) from NHANES, and Asian (*n* = 389) from the Korea NHANES. We also selected 5 times age-, sex-, race-, and survey year-matched general population among participants who had never been diagnosed with cancer (*N* = 4110). Variables were defined using questionnaire data, physical exams, and laboratory tests.

**Results:**

Compared to NHW, Hispanics (aOR 1.15, 95% CI 1.00–1.32) had poor or fair general health, lower education (aOR 1.23, 95% CI 1.07–1.40), and lower household income (aOR 1.16, 95% CI 1.01–1.33). AA survivors were more likely to be non-coupled (aOR 1.35, 95% 1.15–1.60) and have hypertension (aOR 1.18, 95% CI 1.03–1.36). Asians were more former/current drinkers (aOR 1.21, 95% CI 1.05–1.40). NHW are more likely to experience psychological limitation. Compared to matched general, NHW and Asian survivors had poor general health and psychological health.

**Conclusions:**

This study provides evidence for future studies concerning long-term health after AYA cancer survivorship that may vary according to race.

**Supplementary Information:**

The online version contains supplementary material available at 10.1186/s12916-023-03005-3.

## Background

Globally, 1.2 million individuals aged between 15 and 39, which includes adolescents and young adults (AYA), are diagnosed with cancer annually [1]. Disease-free 5-year survival in AYAs is 83–89% across all types of cancers [2]. As survival rates increase, survivors are at risk of long-term health problems; up to 70% report at least one chronic health problem and up to 40% have severe problems and need ongoing medical intervention or surveillance [3, 4]. The period of adolescence and young adulthood is a crucial time for developmental changes socially, physiologically, and psychologically, and the cancer experience in AYA could affect the patient’s long-term life [5].

The current AYA guidelines for survivorship were developed based on studies from the US and European AYA populations [6, 7], but previous studies identified that health-related quality of life in cancer survivors varied according to race [8]. In fact, Asian AYA survivors who live in Asia countries have more unmet needs in communication and information [9], while Western AYA survivors who live in Western countries have more unmet needs regarding support for physical symptoms management [10, 11].

Social, physical health, and psychological health in AYA survivors are associated with multiple genetic, behavioral, environmental, and socioeconomic risk factors, which may vary substantially across racial groups. However, differences in long-term health by race/ethnicity in AYA survivors have not been fully elucidated [12, 13]. Thus, we aimed to compare psychosocial and physical health among AYA survivors by race/ethnicity and their matched general population using nationally representative surveys from the USA and South Korea.

## Methods

### Data source and study participants

**Fig. 1:**
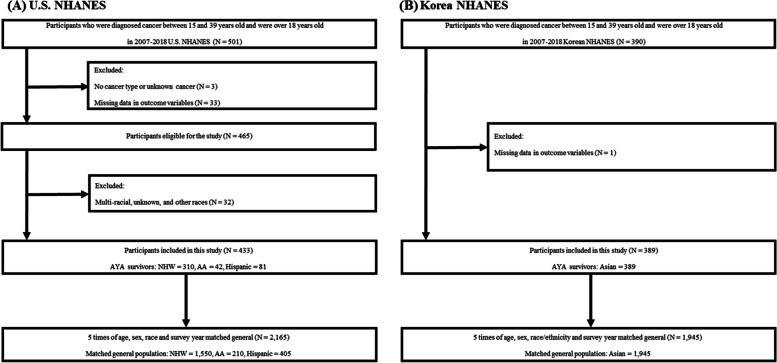
Flow chart of study population. AA, African American; AYA, adolescent and young adult; NHANES, National Health and Nutrition Examination Survey; NHW, non-Hispanic White

We conducted a cross-sectional study using the US National Health and Nutrition Examination Survey (NHANES) and the Korea NHANES (KNHANES) from 2007 to 2018. Both surveys provide nationally representative cross-sectional study of the non-institutionalized population using a multistage cluster sampling design [14, 15]. In both NHANES and KNHANES, each participant completed the questionnaire only once, indicating that each patient received one questionnaire. Both NHANES and KNHANES surveys were conducted in a cross-sectional study every year, each involving a different sample population. The study population included AYA cancer survivors [16, 17]. Participants who were diagnosed with any type of cancer aged between 15 and 39 years and who were adults over 18 years old at survey year were defined as AYA cancer survivors. The NHANES data only categorized race as Non-Hispanic White, African American, and Hispanic, and information on the racial group for participants in the “other” category was not available. As a result, data from other racial categories could not be included. Hence, we excluded participants who were multi-racial, had unknown or other races, had no cancer type or unknown cancer, and had missing data in outcome variables (Fig. 1). Data on participants finally categorized as Non-Hispanic White (NHW, *n* = 310), African American (AA, *n* = 42), or Hispanic (*n* = 81) were obtained from the US NHANES dataset spanning 2007 to 2018.

To supplement the US NHANES data, we obtained data on Asians from the KNHANES dataset, which included a representation sample of Koreans (Asian, *n* = 389). Although the KNHANES dataset only included Koreans, Korean AYA survivors shared similar overall characteristics with other Asian AYA survivors from countries such as Taiwan, Japan, or China [18]. This made it possible to compare the outcomes of Asian AYA survivors with those of other racial groups included in the study (Fig. 1).

We also selected 5 times age at survey year-, sex-, race-, and survey year-matched general population among participants who had never been diagnosed with cancer (*N* = 4110).

### Measurement

#### Cancer type and age at diagnosis

Cancer cases were identified through self-reporting of physician-diagnosed cancer using standardized, self-administered questionnaires. Age at diagnosis and cancer type were also obtained from the self-reported questionnaire. Some types of cancer were written by text; thus, we categorized them. The types of cancer were classified as breast, thyroid, hematologic (lymphoma, leukemia), gynecologic or genitourinary (cervical, ovarian, endometrial, uterine, testicular, kidney, prostate), skin/melanoma, liver, colorectal, gastric, lung, and others. Time since cancer diagnosis was obtained based on the time interval from age at cancer diagnosis to attained age at survey year.

#### General health

General health was assessed using a self-reported question on current general health with a 5-point Likert scale. Participants who reported poor or fair general health were considered to have poor/fair general health.

#### Social health

The social health data included marital status, education level, employment status, average working hours, yearly household income, household type, and smoking and alcohol status from self-reported questionnaire. Marital status was grouped as non-coupled with single, divorced, widowed, and separated, and coupled with married and living with partner. Low education level was defined as participants who reported less than high school graduate. Employment status was classified as unemployed, employed, and self-employed. Low yearly household income was defined as less than $20,000 per year which was used in previous studies with NHANES data [19]. Smoking and alcohol status was classified as never, former, and current smoker or drinker.

#### Physical health

For female participants, reproductive health was measured based on pregnancy and birth experience using a self-reported questionnaire. Hypertension was defined as systolic blood pressure ≥ 140 mmHg, diastolic blood pressure ≥ 90 mmHg, self-reported history of hypertension, or current use of antihypertensive medications. Dyslipidemia was defined as low-density lipoprotein cholesterol level ≥ 130 mg/dL, high-density lipoprotein cholesterol level ≤ 40 mg/dL, self-reported history of dyslipidemia, or current use of lipid-lowering medications [20]. Diabetes mellitus (DM) was defined as a fasting serum glucose level ≥ 126 mg/dL, a self-reported history of DM, or current use of glucose-lowering medications. Body mass index (BMI) and waist circumference were obtained through physical examination. We categorized obesity as BMI ≥ 30.0 kg/m^2^ for NHW, AA, and Hispanic, and BMI ≥ 25.0 kg/m^2^ for Asians according to the World Health Organization guidelines [21]. Other comorbidities, including stroke, angina/angina pectoris, myocardial infarction, arthritis, thyroid disease, and asthma, were defined as self-reported physician diagnoses. Comorbidities were classified as cardiovascular or non-cardiovascular diseases.

#### Psychological health

Psychological health includes daily activity limitations due to emotional problems, depression, and suicide ideation with self-reported questionnaire. Participants who responded yes to daily activity limitations due to emotional problems were defined as poor psychosocial health. Depression was defined as ≥ 10 total scores in PHQ-9 which is a validated measurement [22]. In K-NHANES, PHQ-9 was added after 2014 and was assessed once every 2 years. Hence, we only included PHQ-9 data from 2014 to 2018 in K-NHANES. This approach was also used in the previous article [23]. Suicide ideation was defined as an affirmative answer to the question, “I have thought that I wanted to die at some point in the last year,” or responses to question 9 in the PHQ-9 [24]. Detailed information regarding each study has been published [25].

### Statistical analysis

Since the NHANES and KNHANES data were obtained through multistage-clustered sampling, we analyzed the survey weights for the complex sampling design. Weighted values for each merged dataset were calculated when merging the yearly data. We performed several tests to evaluate differences between AYA’s of different ethnicities. First, we compared different ethnicities among AYA survivors. For the AYA survivor group comparison, we compared the overall difference among the groups. Continuous and categorical variables were compared among the four groups (NHW, AA, Hispanic, and Asian) using weighted analysis of variance (ANOVA) and *χ*^2^ tests, respectively. In addition, for all health, we used logistic regression to estimate adjusted odds ratios (aORs) and 95% confidence intervals (CIs). In regression analysis, we compared race with references NHW by adjusting for age, sex, diagnosed age, survey year, and cancer types. We also compared different ethnicities within the general population using the same methods employed for the AYA group.

To address association differences between AYA and general, we compared the differences between the general population and AYA individuals within each ethnicity. We compared AYA and matched general population within races in continuous and categorical variables using ANOVA and *χ*^2^ tests, respectively. Because the general population were selected using a matching process, we did not perform a weighted analysis. We also used logistic regression to estimate aORs and 95% CIs, comparing each race and ethnicity group to the matched general population, adjusting for age and sex. Additionally, we examined whether the magnitude of the differences between the general population and AYA varied across ethnicities using interaction analysis. We calculated the *p*-value for the interaction to test the significance of the interaction terms between cancer and multiple race groups. Significance in the interaction *p*-values would indicate that different ethnicities could influence the disparities in outcomes between AYA and the general population.

*P* values < 0.05 were considered significant, and two-sided tests were used for all calculations. However, since we had multiple outcomes, we also calculated the Benjamini-Hochberg's adjustment. We start with the largest p-value pk (where p1 ≤ p2≤ … ≤ pk are the p-values of the multiple tests in ascending order) and identify the first i (staring from k) such that pi < (i/k)α. Once this was found then tests 1, ..., i are considered to be significant and the other tests are not significant [26]. The adjusted significant level was 0.042307692. Statistical analyses were performed using R 4.1.2 (R Foundation for Statistical Computing, Vienna, Austria).

## Results

### Study participants

The average age at diagnosis was oldest among Asian survivors (33.0 years) and youngest among Hispanic survivors (28.6 years) (*p* < 0.01). The number of men was highest among NHW survivors (28.5%) and lowest among AA survivors (11.1%) (*p* = 0.02). The most common type of cancer in all races was gynecologic/genitourinary cancer. NHW survivors had higher proportion of skin/melanoma cancer (34.7%) (*p* < 0.01) while Asian survivors had higher proportion of thyroid and stomach cancer (25.2% and 7.0%, respectively) (Table 1). The average time since diagnosis was 19.56, 18.51, 12.76, and 10.57 in NHW, AA, Hispanic, and Asian, respectively. The weighted proportions and means with standard errors are presented in Table 1, additional supplementary Table S1 and Figure S1 (see Additional file 1: Table S1 and Additional file 3: Figure S1).

**Table 1:** Characteristics of AYA survivors by race/ethnicity

Characteristics	NHW Weighted proportion^a^ (SE)	AA Weighted proportion^a^ (SE)	Hispanic Weighted proportion^a^ (SE)	Asian Weighted proportion^a^ (SE)	*p* value
**Age at survey, years**	48.96 (1.09)	48.12 (2.09)	41.32 (1.47)	43.56 (0.68)	** < 0.01**
**Gender**					**0.02**
Male	28.5 (0.01)	11.1 (0.01)	14.1 (0.01)	23.6 (0.01)	
Female	71.5 (0.01)	88.9 (0.01)	85.9 (0.01)	76.4 (0.01)	
**Age at diagnosis**	29.40 (0.41)	29.61 (0.81)	28.57 (0.83)	33.00 (0.37)	** < 0.01**
**Age at diagnosis categories**					
15–25 years	25.6 (0.02)	24.3 (0.01)	31.2 (0.02)	9.2 (0.01)	
26–30 years	30.7 (0.02)	33.5 (0.02)	28.9 (0.02)	12.6 (0.01)	
31–35 years	25.4 (0.02)	26.2 (0.02)	19.8 (0.01)	39.9 (0.02)	
36–39 years	18.3 (0.01)	16.0 (0.01)	20.1 (0.01)	38.3 (0.02)	
**Cancer type**					** < 0.01**
Gynecologic/Genitourinary	40.2 (0.02)	50.5 (0.02)	61.2 (0.02)	32.3 (0.02)	
Skin/Melanoma	34.7 (0.02)	2.4 (0.01)	5.9 (0.01)	0.4 (0.01)	
Thyroid	5.8 (0.01)	4.9 (0.01)	5.6 (0.01)	25.2 (0.02)	
Hematologic	4.2 (0.01)	10.4 (0.01)	5.2 (0.01)	4.8 (0.01)	
Breast	2.7 (0.01)	13.9 (0.01)	7.0 (0.01)	13.0 (0.01)	
Colorectal	2.7 (0.01)	3.5 (0.01)	0.0 (0.00)	3.8 (0.01)	
Liver	0.8 (0.01)	3.5 (0.01)	0.0 (0.00)	0.6 (0.01)	
Lung	0.5 (0.01)	0.0 (0.00)	0.0 (0.00)	0.0 (0.00)	
Stomach	0.0 (0.00)	1.9 (0.01)	0.0 (0.00)	7.0 (0.01)	
Others	8.4 (0.01)	9.0 (0.01)	15.1 (0.01)	12.9 (0.01)	
**Time since diagnosis, years**	19.56 (0.99)	18.51 (1.98)	12.76 (1.18)	10.57 (0.57)	** < 0.01**

*AA* African American, *NHW* Non-Hispanic White

^a^Descriptive analysis with proportion, mean, and standard error (SE)

### General and social health and healthy behavior in AYA survivors by race/ethnicity

Compared to NHW, Hispanics (aOR 1.15, 95% CI 1.00–1.32) had poor or fair general health (Table 2). In the social health domain, Hispanics had lower education (aOR 1.23, 95% CI 1.07–1.40), lower household income (aOR 1.16, 95% CI 1.01–1.33), and less unemployed (aOR 0.89, 95% CI 0.80–0.99) than NHW survivors. AA survivors were more likely to be non-coupled (aOR 1.35, 95% 1.15–1.60, Table 2) than these in NHW survivors. Moreover, Asian survivors more often had lower education (aOR 1.08, 95% CI 1.00–1.16) than NHW survivors. Compared to the NHW, Hispanic and Asian survivors were less likely to be former/current smokers (aOR 0.76, 95% CI 0.66–0.87; aOR 0.76, 95% CI 0.68–0.84, respectively). Hispanic were less likely to be former/current drinkers (aOR 0.82, 95% CI 0.68–0.98) while Asian survivors were more likely to be former/current drinkers (aOR 1.21, 95% CI 1.05–1.40, Table 2). In terms of the general population, we observed a similar trend to that of AYA cancer survivors (Additional file 2: Table S2).

**Table 2:** Adjusted odds ratios and 95% confidence intervals of general and social health, and healthy behavior in AYA survivors by race/ethnicity

	**NHW** **aOR (95% CI)**^a^	**AA** **aOR (95% CI)**^a^	**Hispanic** **aOR (95% CI)**^a^	**Asian** **aOR (95% CI)**^a^
**General health,** *poor/fair*	*reference*	1.09 (0.92–1.28)	1.15 (1.00–1.32)	1.06 (0.96–1.18)
**Social health**				
** Education,** *less than high school graduate*	*reference*	0.99 (0.88–1.11)	1.23 (1.07–1.40)	1.08 (1.00–1.16)
** Marital status*,**** non-coupled*	*reference*	1.35 (1.15–1.60)	1.08 (0.95–1.23)	0.97 (0.88–1.08)
** Yearly household income*,**** less than $20,000*	*reference*	1.10 (0.95–1.27)	1.16 (1.01–1.33)	1.04 (0.97–1.11)
** Current job status, ***unemployed*	*reference*	1.01 (0.85–1.21)	0.89 (0.80–0.99)	1.07 (0.97–1.19)
**Health behavior**				
** Smoking status,** *former/current*	*reference*	0.97 (0.81–1.15)	0.76 (0.66–0.87)	0.76 (0.68–0.84)
** Alcohol status,*** former/current*	*reference*	1.19 (0.88–1.60)	0.82 (0.68–0.98)	1.21 (1.05–1.40)

*AA* African American, *aOR* Adjusted odds ratio, *CI* Confidence interval, *NHW* Non-Hispanic White

^a^Logistic regression with adjusting age, sex, diagnosed age, survey year, and cancer type

### Comorbidities, reproductive and psychological health in AYA survivors by race/ethnicity

AA and Hispanic survivors were more likely to have comorbidities related to cardiovascular disease (CVD), with AA survivors having a higher likelihood of hypertension (aOR 1.18, 95% CI 1.03–1.36) and Hispanic survivors having a higher likelihood of diabetes mellitus (aOR 1.11, 95% CI 1.02–1.20) than NHW survivors (Table 3). Hispanic survivors, however, were less likely to have dyslipidemia (aOR 0.91, 95% CI 0.84–0.99) and arthritis (aOR 0.88, 0.79–0.98) than NHW survivors. Conversely, Asian survivors were less likely to have both CVD and non-CVD comorbidities, with a lower likelihood of hypertension (aOR 0.91, 95% CI 0.83–0.99), stroke (aOR 0.97, 95% CI 0.94–0.99), myocardial infarction (aOR 0.96, 95% CI 0.93–0.99), dyslipidemia (aOR 0.89, 95% CI 0.83–0.96), arthritis (aOR 0.75, 95% CI 0.69–0.82), thyroid disease (aOR 0.85, 95% CI 0.78–0.93), and asthma (aOR 0.79, 95% CI 0.73–0.87) compared to NHW AYA survivors.

**Table 3:** Adjusted odds ratios and 95% confidence intervals of comorbidities, reproductive and psychological health in AYA survivors by race/ethnicity

	**NHW** **aOR (95% CI)**^a^	**AA** **aOR (95% CI)**^a^	**Hispanic** **aOR (95% CI)**^a^	**Asian** **aOR (95% CI)**^a^
**Comorbidities**				
**Cardiovascular disease**				
Hypertension	*reference*	1.18 (1.03–1.36)	0.95 (0.86–1.04)	0.91 (0.83–0.99)
Stroke	*reference*	1.09 (0.99–1.20)	0.99 (0.95–1.03)	0.97 (0.94–0.99)
Angina/angina pectoris	*reference*	1.00 (0.93–1.06)	0.96 (0.93–1.00)	0.97 (0.93–1.01)
Myocardial infarction	*reference*	1.00 (0.93–1.08)	0.98 (0.94–1.02)	0.96 (0.93–0.99)
Obesity	*reference*	1.14 (0.98–1.32)	1.11 (0.96–1.28)	0.94 (0.84–1.06)
Dyslipidemia	*reference*	0.99 (0.87–1.13)	0.91 (0.84–0.99)	0.89 (0.83–0.96)
DM	*reference*	1.06 (0.97–1.15)	1.11 (1.02–1.20)	1.03 (0.98–1.09)
** Non-cardiovascular disease**				
Arthritis	*reference*	1.03 (0.88–1.20)	0.88 (0.79–0.98)	0.75 (0.69–0.82)
Thyroid disease	*reference*	0.97 (0.86–1.08)	1.02 (0.92–1.13)	0.85 (0.78–0.93)
Asthma	*reference*	0.89 (0.77–1.04)	0.97 (0.85–1.10)	0.79 (0.73–0.87)
**Reproductive health**^**b**^				
History of pregnant, *yes*	*reference*	1.08 (0.96–1.21)	1.08 (0.96–1.21)	1.00 (0.90–1.10)
History of birth, *yes*	*reference*	1.05 (0.91–1.21)	1.08 (0.96–1.22)	0.99 (0.90–1.10)
**Psychological health**				
**Daily activity limitation due to emotional problem, *yes***	*reference*	0.75 (0.39–1.42)	0.77 (0.46–1.28)	0.65 (0.47–0.88)
Depression, *PHQ-9* ≥ *10*^**c**^	*reference*	1.00 (0.95–1.07)	0.97 (0.94–1.01)	1.07 (0.99–1.17)
Suicide ideation, *yes*	*reference*	0.97 (0.93–1.06)	0.99 (0.93–1.06)	1.07 (1.01–1.14)

*AA* African American, *aOR* Adjusted odds ratio, *CI* Confidence interval, *DM* Diabetes mellitus, *NHW* Non-Hispanic White

^a^Logistic regression with adjusting age, sex, diagnosed age, survey year, and cancer type

^b^Female only

^c^Only include available PHQ-9 data in NHANES from 2007 to 2018 and K-NHANES from 2014 to 2018

Reproductive health was similar among the AYA groups (Table 3). In terms of psychological health, Asian were more likely to experience suicide ideation (aOR 1.07, 95% CI 1.01–1.14) than NHW (Table 3). In terms of the general population, we observed a similar trend to that of AYA cancer survivors (Additional file 2: Table S2).

### Social, physical, and psychological health in AYA survivors and matched general by race/ethnicity

Compared to matched general by race/ethnicity, NHW and Asian survivors were more likely to have poor or fair general health (aOR 1.59, 95% CI 1.17–2.15; aOR 2.93, 95% CI 2.27–3.77, respectively, Table 4). NHW survivors were more likely to be non-coupled (aOR 1.33, 95% CI 1.04–1.71) and unemployed (aOR 1.34, 95% CI 1.03–1.73) than their matched general. Moreover, NHW survivors were more former/current smokers (aOR 1.62, 95% CI 1.26–2.07), while AA were more former/current drinkers (aOR 5.51, 95% CI 1.34–37.53) than the matched general (Table 4).

**Table 4:** Interaction analysis of social, physical, and psychological health of AYA survivors compared to the matched general population by race/ethnicity (*N* = 4932)

	**AYA vs. age, sex, year, and race matched**^a^** general**				***P for interaction***
	**NHW** **aOR (95% CI)**^a^	**AA** **aOR (95% CI)**^a^	**Hispanic** **aOR (95% CI)**^a^	**Asian** **aOR (95% CI)**^a^	
**General health,** *poor/fair*	1.59 (1.17–2.15)	1.30 (0.58–2.78)	1.35 (0.79–2.30)	2.93 (2.27–3.77)	** < 0.01**
**Social health**					
**Education,** *less than high school graduate*	1.14 (0.81–1.58)	0.71 (0.25–1.73)	0.63 (0.38–1.03)	1.13 (0.80–1.59)	0.07
**Marital status*,**** non-coupled*	1.33 (1.04–1.71)	1.15 (0.58–2.37)	1.49 (0.91–2.44)	0.95 (0.73–1.22)	0.14
**Yearly household income*,**** less than $20,000*	1.09 (0.80–1.46)	0.97 (0.44–2.03)	1.29 (0.75–2.18)	1.02 (0.74–1.38)	0.82
**Current job status, ***unemployed*	1.34 (1.03–1.73)	1.38 (0.69–2.75)	0.63 (0.37–1.06)	1.10 (0.87–1.38)	0.08
**Physical health**					
**Health behavior**					
Smoking status, *former/current*	1.62 (1.26–2.07)	1.79 (0.90–3.58)	0.89 (0.49–1.57)	0.92 (0.67–1.24)	** < 0.01**
Alcohol status,* former/current*	1.27 (0.73–2.26)	5.51 (1.34–37.53)	1.16 (0.54–2.50)	0.90 (0.63–1.31)	** < 0.01**
**Reproductive health**^b^					
**History of pregnant, *yes***	1.48 (0.94–2.44)	1.18 (0.29–7.99)	1.17 (0.42–4.13)	1.56 (0.97–2.62)	0.89
**History of birth, *yes***	1.48 (0.94–2.42)	0.76 (0.21–3.54)	1.15 (0.42–4.08)	1.73 (1.11–2.80)	0.60
**Comorbidities**					
**Cardiovascular**					
Hypertension	1.34 (0.99–1.80)	1.78 (0.77–4.16)	1.32 (0.68–2.50)	0.79 (0.56–1.08)	**0.04**
Stroke	1.55 (0.86–2.68)	3.21 (1.02–9.44)	2.53 (0.52–10.01)	1.26 (0.28–4.09)	** < 0.01**
Angina/angina pectoris	1.54 (0.80–2.78)	1.93 (0.27–9.36)	0.63 (0.03–3.97)	3.92 (1.27–11.54)	0.69
Myocardial infarction	1.93 (1.11–3.26)	1.91 (0.27–9.36)	1.42 (0.21–6.05)	3.88 (0.75–18.02)	0.20
Obesity	0.88 (0.68–1.14)	0.79 (0.40–1.57)	1.11 (0.68–1.82)	1.08 (0.84–1.38)	0.59
Dyslipidemia	1.52 (0.99–1.80)	1.27 (0.77–4.16)	1.06 (0.68–2.50)	1.64 (0.56–1.08)	0.53
DM	1.35 (0.87–2.06)	1.16 (0.41–2.96)	1.67 (0.81–3.27)	1.31 (0.85–1.98)	0.72
**Non-cardiovascular**					
Arthritis	1.82 (1.39–2.38)	2.67 (1.26–5.72)	2.49 (1.35–4.56)	1.14 (0.75–1.70)	** < 0.01**
Thyroid disease	1.31 (0.93–1.83)	1.89 (0.69–4.71)	3.81 (2.06–6.97)	2.57 (1.73–3.76)	** < 0.01**
Asthma	1.63 (1.22–2.18)	0.79 (0.31–1.83)	2.65 (1.42–4.82)	1.92 (1.09–3.23)	** < 0.01**
**Psychological health**					
Daily activity limitation due to emotional problem, *yes*	0.83 (0.29–2.42)	1.32 (0.04–32.88)	0.30 (0.06–1.41)	2.07 (0.77–5.61)	0.25
Depression, *PHQ-9* ≥ *10*^**c**^	2.63 (1.21–5.41)	1.03 (0.05–7.37)	0.73 (0.04–4.23)	2.34 (0.99–5.16)	0.23
Suicide ideation, *yes*	2.01 (1.14–3.41)	1.16 (0.23–7.31)	1.34 (0.38–3.82)	1.47 (1.01–2.08)	0.92

*AA* African American, *aOR* Adjusted odds ratio, *CI* Confidence interval, *DM* Diabetes mellitus, *NHW* Non-Hispanic White

^a^Logistic regression with adjusting age and sex, reference group: AYA matched general by race/ethnicity

^b^Female only

^c^Only include available PHQ-9 data in NHANES from 2007 to 2018 and K-NHANES from 2014 to 2018

Asian survivors were also more likely to have a history of birth (aOR 1.73, 95% CI 1.11–2.80) than matched general. NHW survivors were more likely to have myocardial infarction (aOR 1.93, 95% CI 1.11–3.26), arthritis (aOR 1.82, 95% CI 1.39–2.38), and asthma (aOR 1.63, 95% CI 1.22–2.18) than matched general. AA had more stroke (aOR 3.21, 95% CI 1.02–9.44) and arthritis (aOR 2.67, 95% CI 1.26–5.72) compared to matched general. Hispanics more likely to have arthritis (aOR 2.49, 95% CI 1.35–4.56), thyroid disease (aOR 3.81, 95% CI 2.06–6.97), and asthma (aOR 2.65, 95% CI 1.42–4.82) than matched general. Asian survivors had more angina/angina pectoris (aOR 3.92, 95% CI 1.27–11.54), thyroid disease (aOR 2.57, 95% CI 1.73–3.76), and asthma (aOR 1.92, 95% CI 1.09–3.23) than matched general (Table 4).

**Fig. 2:**
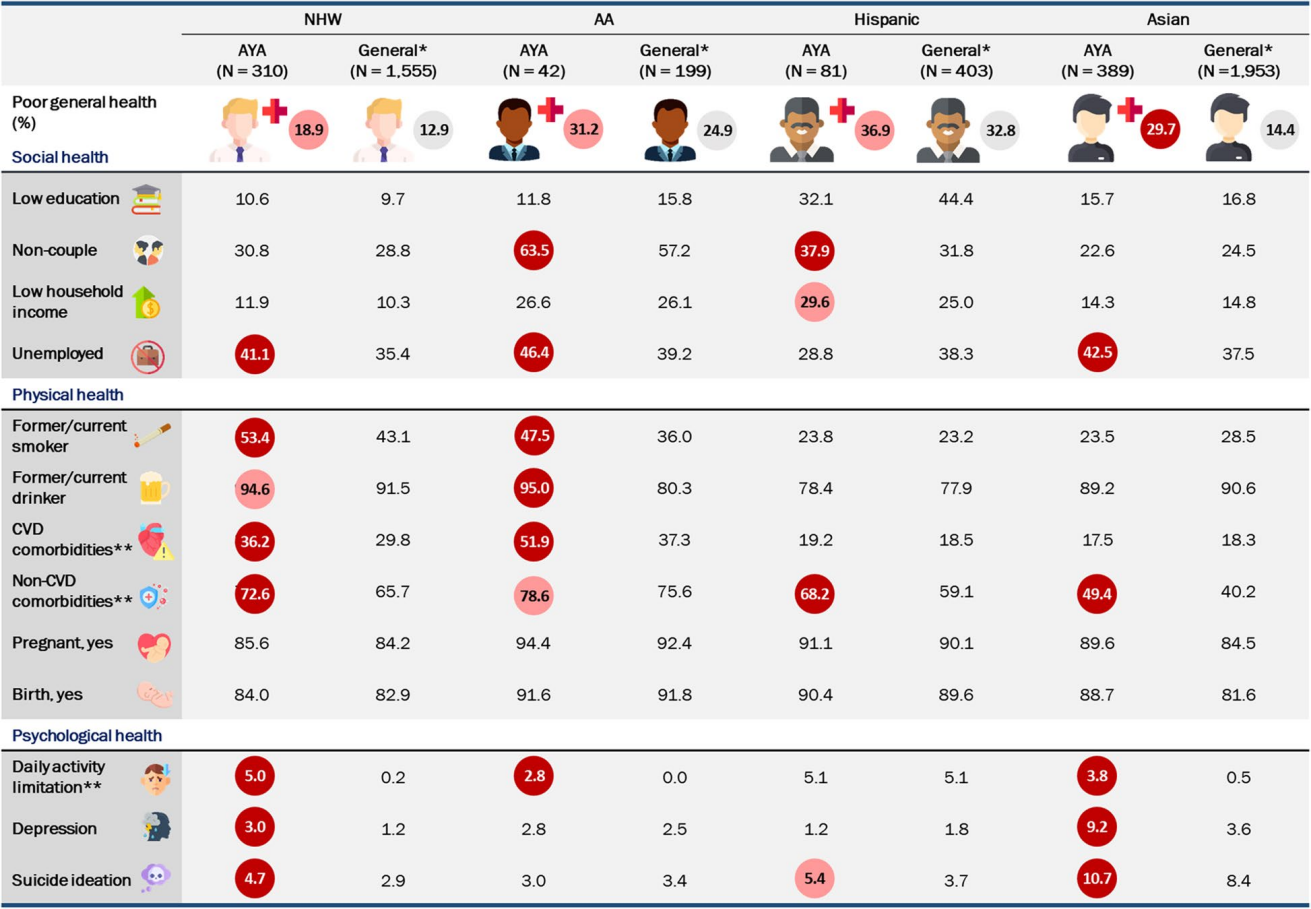
Prevalence of social, physical, and psychological health characteristics in AYA survivors and matched general by race/ethnicity. AA, African American; NHW, non-Hispanic White. General: age, sex, and survey year matched general population by race/ethnicity group. *Descriptive analysis with proportion (%) and the greatest value was highlighted with red circle. ^**^Cardiovascular comorbidities: hypertension, stroke, angina/angina pectoris, myocardial infarction, obesity, diabetes mellitus, dyslipidemia; non-cardiovascular comorbidities: arthritis, thyroid disease, asthma; daily limitation: daily activity limitation due to emotional problem

NHW survivors had more experiences with depression (aOR 2.63, 95% CI 1.21–5.41) and both NHW and Asian survivors had more suicide ideation (aOR 2.01, 95% CI 1.14–3.41; aOR 1.47, 95% CI 1.01–2.08, respectively) compared to their matched general (Table 4). The weighted proportions and means with standard errors among matched general are also presented in Fig. 2 and additional supplementary Table S2 (see Additional file 2: Table S2).

## Discussion

In this multinational study, we found differences in type of cancer and social, physical, and psychological health among race and ethnicity groups in AYA survivors. Compared to NHW survivors, Hispanic survivors were more likely to report poor general health and lowest levels of education and household income. AA survivors were more likely to be non-coupled. NHW and Asian survivors were most likely to have poor psychological health among the races. Compared to their matched general, NHW and Asian survivors had poor general health and psychological health.

In previous studies, the most common cancers in AYA survivors were thyroid cancer, followed by leukemia and non-Hodgkin lymphoma, skin/melanoma, breast cancer, and cervical cancer [2, 27]. However, we found that the frequency of cancer type differed by race among the AYA cancer survivors. More than half of the Hispanic and AA patients have experienced gynecologic/genitourinary cancer in our study. Gynecologic/genitourinary cancer, especially cervical cancer, is the second leading cause of cancer-related death among AYA women in the USA; however, the mortality rate has markedly decreased over the past few decades [28] due to the availability of human papillomavirus vaccination and the adoption of cervical screening. Thus, gynecologic/genitourinary cancers may have the largest proportion among survivors. The Asian AYA had the highest prevalence of stomach cancer compared to the other race/ethnic groups. The prevalence of *Helicobacter pylori* infection is higher in Asia and South America than that in the USA. A study assessing racial/ethnic differences found that 31.4% of gastric cancers in Asian Americans occurred in the pyloric antrum compared with 19.6% for all races [29]. Consistent with the possibility that endemic infection and cultural factors contribute to risk, several studies have shown that migration from high- to low-incidence regions, such as from Asia to the USA, is associated with a decreased risk of developing stomach cancer [29].

In social health, Hispanic survivors had the lowest education level and lowest income in this study. According to previous studies, Hispanic survivors were more likely to be diagnosed with cancer at younger ages than other races [30], and it is strongly related to the interruption of education [31], which led to lower income levels. AA survivors were more non-coupled in our study. Previous studies had consistent result that cancer survivors were more likely to be divorced and separated due to the burden from cancer treatment, emotional distress, economic hardship, and infertility [32]. Further studies are required to identify long-term marriage and relationship effects from cancer and treatment whether someone marries or ends their marriage in AYA survivors.

In this study, it was found that AA and Hispanic survivors had a higher likelihood of comorbidities related to CVD, but a lower likelihood of non-CVD-related comorbidities compared to NHW. In general, AAs and Hispanics in the USA have higher BMI and prevalence of obesity than NHW and Asian populations [33]. Among cancer survivors, AA and Hispanics also had more than two times higher prevalence of obesity and obesity-related disease. Notably, Hispanic AYA survivors were more likely to have DM compared with the other races in our study. The prevalence of both diagnosed and undiagnosed type 2 DM is nearly twice as high among Mexican-origin Hispanic/Latino adults compared to NHWs in the USA [34]. Furthermore, AA survivors were more likely to have hypertension than NHW survivors, which is similar to previous studies [35]. Hypertension was also associated with BMI [35]. On the other hand, Asians had the lowest comorbidity. It was a similar pattern, with a lower prevalence of comorbidities, which has been observed in other Asian countries [18]. The prevalence of CVD-related comorbidities among Asian cancer survivors was 3.5–16% [18]. In particular, the low prevalence of myocardial infarction in Asians may reflect lower background rates of the disease owing to environmental or genetic factors [25]. The prevalence of comorbidities varies by race and ethnicity; hence, future studies and guidelines need to consider race and ethnicity in physical health.

In terms of psychological health, we found that NHW and Asian survivors had the worst psychological health with experiences of depression and suicide ideation. Psychological burden is a common late- and long-term effect in patients with cancer [36]. Self-inflicted injury is the second most common cause of death among individuals aged 15 and 39 years [37]. However, a limited number of previous studies have examined racial disparities in the psychological burden of AYA cancer in long-term survivors [36]. Hence, additional research on racial disparities in the psychological burden is required.

Compared to matched general by race/ethnicity, AYA survivors were least likely to receive education and unemployed compared with the general population. AYA includes physical, cognitive, emotional, and social transitions to achieve developmental milestones, like finding a job, becoming financially independent, forming relationships, and starting a family [38]. However, a cancer diagnosis and treatment interruption and delay impede the achievement of these personal goals both in the short and long terms [39]. Additionally, NHW survivors had a higher rate of former/current smokers than other races and matched general. According to the Tobacco Use Supplements to the Current Population Surveys, smoking prevalence and cigarette consumption levels have been historically higher in general among NHW than among AA and Hispanics [40]. Although the prevalence of tobacco use has continued to decline in highly educated NHW over the past three decades, less educated NHW have remained at risk [40]. These findings are in line with the minorities’ diminished returns theory [41], which postulates that NHW may experience a huge decreasing protective effect with decreasing educational attainment than racial/ethnic minorities.

The limitation in returning to a normal life could affect depression and suicide ideation as well as poor general health in AYA cancer survivors more than in their matched general population. Especially, Asian AYA survivors had much higher prevalence of suicide ideation than matched general in this study. According to a national survey in Korea, more than half of the public had negative attitudes, stereotypes, and discrimination toward cancer patients in spite of medical advancements and improved survival rate [42]. Such an unfavorable environment would make it difficult for Korean cancer AYA survivors to return to work after cancer treatment resulting in poor quality of life [43, 44].

Several limitations should be considered when interpreting our findings. First, we used a cross-sectional study and did not have information on the timing of the development of each variable. However, our objective was to compare the current patterns of social, physical, and psychological health across race/ethnic groups and not to identify causal pathways. Second, we used self-reported questionnaire data, which might have led to recall bias. According to a previous study, when compared with confirmed cancer in the national cancer registry data, the sensitivity and specificity for self-reporting of physician-diagnosed breast cancer were 97.1% and 99.1%, respectively [23]. Moreover, to reduce the recall bias, a shorter recall period is effective [45]. Our study compared the current status of health; hence, we mostly used questions about the current status. Third, our study included only AYA survivors, without treatment types. However, this study aimed to analyze the current health status of survivors. Fourth, the different patterns in health may be due to different cultural and environmental exposures between USA and Korea. However, race/ethnicity is also a risk factor for health status and disparities of cultural and environmental background in different race/ethnicity might be also contributed to different cultural and environmental exposures which could be affected from racial differences in health [46]. Furthermore, despite Korean AYA survivors exhibiting overall similar characteristics to AYA survivors from other Asian countries such as Taiwan, Japan, or China, there are still variations attributed to differences in healthcare systems. Therefore, future research should focus on broader samples of Asian AYA survivors to enhance the generalizability of the results. Lastly, while it was not our intention to exclude AYA survivors with more severe long-term health issues, there is a possibility that their inclusion was limited in our study. AYA cancer survivors who are still undergoing active treatment and experiencing severe illness may face unique challenges and considerations that fall outside the scope of our research. Therefore, further studies specifically targeting this population are necessary to gain a better understanding of their experiences, outcomes, and support needs.

## Conclusions

We identified multidimensional relationships between physical, psychological, and social health statuses by race and ethnicity. Racial differences in socioeconomic status are important contributors to racial health disparities [46]. Individuals from racial/ethnic minorities may be exposed to socio-environmental conditions and stressors that affect health throughout life [46]. In addition, we identified relationships between health and long-term survival of more than 10 years. This study provides evidence for future studies concerning long-term health that may vary according to race and ethnicity. Therefore, we believe that race and ethnicity should be considered to improve the overall health status of AYA cancer survivors.

### Supplementary Information

**Additional file 1: Supplement Table S1.** Prevalence of social, physical, and psychological health characteristics in AYA survivors by race/ethnicity. The weighted proportion and standard error by race/ethnicity group in AYA survivors in our study.

**Additional file 2: Supplement Table S2.** Prevalence of social, physical, and psychological health characteristics in matched general by race/ethnicity. The weighted proportion and standard error by race/ethnicity group in matched general in our study.

**Additional file 3: Supplement Figure S1.** Prevalence of social, physical, and psychological health characteristics in AYA survivors by race/ethnicity. The weighted proportion of AYA survivors by race/ethnicity group in our study. The greatest values were highlighted with red circle. ^**^ Cardiovascular comorbidities: hypertension, stroke, angina/angina pectoris, myocardial infarction, obesity, diabetes mellitus, dyslipidemia; Non-cardiovascular comorbidities: arthritis, thyroid disease, asthma; Daily limitation: daily activity limitation due to emotional problem.

## Data Availability

All data extracted in this study are included in this article.

## Abbreviations

AA: African American
ANOVA: Analysis of variance
aOR: Adjusted odds ratio
AYA: Adolescent and young adult
BMI: Body mass index
CIs: Confidence intervals
CVD: Cardiovascular disease
DM: Diabetes mellitus
KNHANES: The Korean National Health and Nutrition Examination Survey
NHANES: The National Health and Nutrition Examination Survey
NHW: Non-Hispanic White
