# Supplementary material for: Racial differences in long-term social, physical, and psychological health among adolescent and young adult cancer survivors
Source: BMC Med. 2023 Aug 4;21:289. doi: 10.1186/s12916-023-03005-3 (PMC10403852; doi:10.1186/s12916-023-03005-3)
Supplement: Supplementary file 1 — Additional file 1: Supplement Table S1. Prevalence of social, physical, and psychological health characteristics in AYA survivors by race/ethnicity. The weighted proportion and standard error by race/ethnicity group in AYA survivors in our study. [file 12916_2023_3005_MOESM1_ESM.pdf]

Supplement Table S1. Prevalence of social, physical, and psychological health characteristics in AYA survivors by race/ethnicity

Characteristics	NHW weighted proportion* (SE)	AA weighted proportion* (SE)	Hispanic weighted proportion* (SE)	Asian weighted proportion*(SE)	P value	a§
**General health**					**0.03**	0.04
Poor	2.9 (0.03)	10.7 (0.01)	8.0 (0.04)	7.6 (0.01)		
Fair	16.0 (0.06)	20.5 (0.01)	28.9 (0.07)	22.1 (0.01)		
Good	45.4 (0.08)	43.7 (0.02)	38.2 (0.08)	45.4 (0.02)		
Very good	26.3 (0.07)	21.1 (0.01)	18.8 (0.06)	21.4 (0.01)		
Excellent	9.4 (0.05)	4.0 (0.01)	6.1 (0.04)	3.5 (0.01)		
**Social health**						
**Education**					**< 0.01**	<0.01
Less than high school graduate	10.6 (0.01)	11.8 (0.01)	32.1 (0.02)	15.7 (0.01)		
High school graduate	16.6 (0.01)	15.7 (0.01)	25.2 (0.02)	40.0 (0.02)		
More than university	72.8 (0.02)	72.5 (0.02)	42.7 (0.02)	44.3 (0.02)		
**Current job status**					**< 0.01**	<0.01
Unemployed	41.1 (0.10)	46.4 (0.02)	28.8 (0.09)	42.5 (0.02)		
Employee	54.3 (0.10)	53.6 (0.02)	58.4 (0.10)	46.7 (0.02)		
Self-employed	4.6 (0.04)	0.0 (0.00)	12.8 (0.07)	10.8 (0.01)		
**Employment hours, mean (SE)**	42.94 (1.21)	33.39 (2.97)	34.66 (1.48)	38.10 (1.39)	**< 0.01**	<0.01
**Marital status**					**< 0.01**	<0.01
Single	8.7 (0.01)	28.0 (0.02)	7.5 (0.01)	11.0 (0.01)		
Divorced or separated	16.6 (0.01)	22.0 (0.01)	27.6 (0.02)	7.3 (0.01)		
Widowed	5.5 (0.01)	13.5 (0.01)	2.8 (0.01)	4.3 (0.01)		
Married or living with partner	69.2 (0.02)	36.5 (0.02)	62.1 (0.02)	77.4 (0.01)		
**Yearly household income**					**< 0.01**	<0.01
Less than $20,000	11.9 (0.01)	26.6 (0.02)	29.6 (0.02)	14.3 (0.01)		
$20,000 to $54,999	28.3 (0.02)	34.7 (0.02)	39.8 (0.02)	47.3 (0.02)		

$55,000 to $74,999	16.7 (0.01)	17.4 (0.01)	10.8 (0.01)	19.0 (0.01)		
More than $75,000	43.1 (0.02)	21.3 (0.01)	19.8 (0.01)	19.3 (0.01)		
**Household type**					**0.01**	0.01
Single (living alone)	13.5 (0.01)	11.3 (0.01)	4.6 (0.01)	6.2 (0.01)		
**Physical health**						
**Smoking status**					< **0.01**	0.01
Never	46.6 (0.02)	52.5 (0.02)	76.2 (0.01)	76.5 (0.01)		
Former	26.6 (0.02)	28.2 (0.02)	12.0 (0.01)	11.7 (0.01)		
Current	26.8 (0.02)	19.3 (0.01)	11.8 (0.01)	11.8 (0.01)		
**Alcohol status**					< **0.01**	0.02
Never	5.5 (0.02)	5.0 (0.01)	21.6 (0.02)	10.8 (0.01)		
Former	13.0 (0.02)	26.3 (0.01)	22.6 (0.02)	15.1 (0.01)		
Current	81.6 (0.02)	68.7 (0.02)	55.8 (0.02)	74.1 (0.02)		
**BMI (kg/m^2), mean (SE)**	29.17 (0.56)	30.85 (0.89)	30.56 (0.64)	23.39 (0.21)	< **0.01**	0.02
**Waist circumference (cm), mean (SE)**	99.71 (1.23)	98.68 (2.07)	98.89 (1.53)	79.03 (0.60)	< **0.01**	0.02
**Reproductive health**┼						
Ever pregnant, *yes*	85.6 (0.01)	94.4 (0.01)	91.1 (0.01)	89.6 (0.01)	0.230	0.048
Ever birth, *yes*	84.0 (0.01)	91.6 (0.01)	90.4 (0.01)	88.7 (0.01)	0.760	0.050
**Comorbidities, *yes***						
**Cardiovascular**						
Hypertension	32.7 (0.02)	45.9 (0.02)	17.8 (0.01)	16.4 (0.01)	< **0.01**	0.02
Stroke	3.3 (0.01)	13.1 (0.01)	2.7 (0.01)	0.5 (0.01)	**0.02**	0.039
Angina/angina pectoris	3.6 (0.01)	3.9 (0.01)	0.4 (0.01)	1.2 (0.01)	0.08	0.046
Myocardial infraction	3.8 (0.01)	4.8 (0.01)	2.1 (0.01)	0.4 (0.01)	**0.01**	0.03
Obesity	35.9 (0.02)	50.0 (0.02)	49.6 (0.02)	31.3 (0.02)	**0.01**	0.04
Dyslipidemia	27.8 (0.01)	21.0 (0.01)	9.7 (0.01)	8.1 (0.01)	< **0.01**	0.02
DM	8.3 (0.01)	12.3 (0.01)	14.0 (0.01)	6.6 (0.01)	< **0.01**	0.02

**Non-cardiovascular**						
Arthritis	39.6 (0.02)	43.7 (0.02)	23.4 (0.01)	8.4 (0.01)	< **0.01**	0.02
Thyroid disease	18.6 (0.01)	16.5 (0.01)	20.9 (0.01)	10.7 (0.01)	0.05	0.04
Asthma	23.5 (0.01)	15.2 (0.01)	25.7 (0.02)	3.3 (0.01)	< **0.01**	0.02
**Psychological health**						
Daily activity limitation due to emotional problem, *yes*	5.0 (0.01)	2.8 (0.02)	5.1 (0.02)	3.8 (0.02)	**<0.01**	0.03
Depression, *PHQ-9 ≥10*┼┼	3.0 (0.01)	2.8 (0.01)	1.2 (0.01)	9.2 (0.01)	**0.02**	0.04
Suicide ideation, *yes*	4.7 (0.01)	3.0 (0.01)	5.4 (0.01)	10.7 (0.01)	**<0.01**	0.03

AA, African American; DM, Diabetes mellitus; NHW, non-Hispanic White.

*Weighted analysis of variance (ANOVA) and χ^2 tests for weighted proportion and standard error

┼ Female only

┼┼ Only include available PHQ-9 data in NHANES from 2007-2018 and K-NHANES from 2014-2018.

§Those fields provide the corrected significance level after Benjamini and Hochberg (1995). All p-values below the provided value of “a” show significant results, given the setup you have chosen for multiple testing.
