# Supplementary material for: Racial differences in long-term social, physical, and psychological health among adolescent and young adult cancer survivors
Source: BMC Med. 2023 Aug 4;21:289. doi: 10.1186/s12916-023-03005-3 (PMC10403852; doi:10.1186/s12916-023-03005-3)
Supplement: Supplementary file 2 — Additional file 2: Supplement Table S2. Prevalence of social, physical, and psychological health characteristics in matched general by race/ethnicity. The weighted proportion and standard error by race/ethnicity group in matched general in our study. [file 12916_2023_3005_MOESM2_ESM.pdf]

Supplement Table S2. Prevalence of social, physical, and psychological health characteristics among matched general by race/ethnicity

Characteristics	NHW weighted proportion (SE)	AA weighted proportion (SE)	Hispanic weighted proportion (SE)	Asian weighted proportion (SE)	P value
**General health**					**<0.001**
Poor	11.7 (0.02)	9.4 (0.01)	6.4 (0.02)	4.5 (0.00)	
Fair	39.0 (0.01)	19.0 (0.03)	18.5 (0.01)	31.0 (0.03)	
Good	36.5 (0.03)	46.7 (0.01)	42.2 (0.03)	50.1 (0.01)	
Very good	10.9 (0.01)	19.9 (0.03)	29.1 (0.01)	12.5 (0.02)	
Excellent	2.0 (0.01)	5.0 (0.00)	3.7 (0.01)	1.9 (0.00)	
**Social health**					
**Education**					**<0.001**
Less than high school graduate	9.7 (0.01)	15.8 (0.01)	44.4 (0.01)	16.8 (0.01)	
High school graduate	22.9 (0.01)	25.9 (0.01)	18.7 (0.01)	35.3 (0.01)	
More than university	67.4 (0.01)	58.3 (0.01)	36.9 (0.01)	47.9 (0.01)	
**Current job status**					**<0.001**
Unemployed	35.6 (0.06)	39.4 (0.01)	38.6 (0.06)	38.6 (0.01)	
Employee	57.7 (0.01)	57.1 (0.06)	56.8 (0.01)	47.0 (0.01)	
Self-employed	6.7 (0.03)	3.5 (0.00)	4.6 (0.03)	14.5 (0.01)	
**Employment hours, mean (SE)**	38.52 (14.26)	37.70 (14.04)	39.47 (14.23)	39.94 (17.09)	0.17
**Marital status**					**<0.001**
Single	11.2 (0.01)	28.0 (0.01)	13.6 (0.01)	15.8 (0.01)	
Divorced or separated	12.8 (0.01)	20.4 (0.01)	16.8 (0.01)	4.5 (0.00)	
Widowed	4.8 (0.00)	8.8 (0.01)	1.3 (0.02)	4.2 (0.00)	
Married or living with partner	71.2 (0.01)	42.8 (0.01)	68.2 (0.01)	75.5 (0.01)	
**Yearly household income**					**<0.001**

Less than $20,000	10.3 (0.01)	26.1 (0.01)	25.0 (0.01)	14.8 (0.01)	
$20,000 to $54,999	30.1 (0.01)	43.0 (0.01)	47.1 (0.01)	47.6 (0.01)	
$55,000 to $74,999	11.8 (0.01)	11.0 (0.01)	9.5 (0.01)	15.6 (0.01)	
More than $75,000	47.8 (0.01)	19.8 (0.01)	18.3 (0.01)	22.0 (0.01)	
**Household type**					**<0.001**
Single (living alone)	12.4 (0.01)	12.6 (0.01)	2.8 (0.00)	6.0 (0.00)	
**Physical health**					
**Smoking status**					**<0.001**
Never	56.9 (0.29)	64.0 (0.01)	76.8 (0.24)	71.5 (0.01)	
Former	43.1 (0.01)	36.0 (0.28)	23.2 (0.01)	10.2 (0.17)	
Current	0.0 (0.00)	0.0 (0.00)	0.0 (0.00)	18.2 (0.01)	
**Alcohol status**					**<0.001**
Never	8.5 (0.02)	19.7 (0.01)	22.1 (0.03)	9.4 (0.01)	
Former	11.7 (0.01)	17.8 (0.02)	18.6 (0.01)	13.8 (0.02)	
Current	79.7 (0.03)	62.5 (0.01)	59.3 (0.03)	76.8 (0.01)	
**BMI (kg/m^2), mean**	29.34 (7.33)	32.67 (8.76)	30.17 (6.42)	23.31 (3.45)	**<0.001**
**Waist circumference (cm), mean**	99.66 (17.03)	102.56 (16.97)	98.55 (15.21)	78.38 (9.87)	**<0.001**
**Reproductive health**†					
Ever pregnant, *yes*	84.2 (0.01)	92.4 (0.01)	90.1 (0.01)	84.5 (0.01)	0.07
Ever birth, *yes*	82.9 (0.01)	91.8 (0.01)	89.6 (0.01)	81.6 (0.01)	**0.02**
**Comorbidities, *yes***					
**Cardiovascular**					
Hypertension	28.1 (0.01)	37.0 (0.01)	16.5 (0.01)	17.8 (0.01)	**<0.001**
Stroke	2.5 (0.00)	4.1 (0.00)	1.2 (0.00)	0.5 (0.00)	**<0.001**
Angina/angina pectoris	2.1 (0.00)	2.2 (0.00)	1.4 (0.00)	0.4 (0.00)	**<0.001**
Myocardial infarction	2.2 (0.00)	2.2 (0.00)	1.8 (0.00)	0.2 (0.00)	**<0.001**

Obesity	36.0 (0.01)	59.2 (0.01)	44.8 (0.01)	28.6 (0.01)	**<0.001**
Dyslipidemia	19.1 (0.01)	16.2 (0.01)	7.9 (0.01)	4.8 (0.00)	**<0.001**
DM	7.1 (0.01)	11.3 (0.01)	8.9 (0.01)	5.7 (0.00)	**0.03**
**Non-cardiovascular**					
Arthritis	27.7 (0.01)	25.3 (0.01)	14.4 (0.01)	7.6 (0.01)	**<0.001**
Thyroid disease	13.2 (0.01)	9.1 (0.01)	9.1 (0.01)	4.5 (0.00)	**<0.001**
Asthma	16.7 (0.01)	20.1 (0.01)	10.1 (0.01)	2.9 (0.00)	**<0.001**
**Psychological health**					
Daily activity limitation due to emotional problem, *yes*	0.2 (0.00)	0.0 (0.00)	0.0 (0.00)	0.5 (0.00)	0.63
Depression, *PHQ-9 ≥10*ǂǂ	1.2 (0.00)	2.5 (0.00)	1.8 (0.00)	3.6 (0.00)	**0.001**
Suicide ideation, *yes*	2.9 (0.00)	3.4 (0.00)	3.7 (0.00)	8.4 (0.00)	**<0.001**

AA, African American; DM, Diabetes mellitus; NHW, non-Hispanic White.

ǂ Female only

ǂǂ Only include available PHQ-9 data in NHANES from 2007-2018 and K-NHANES from 2014-2018.
