# Supplementary material for: Racial differences in long-term social, physical, and psychological health among adolescent and young adult cancer survivors
Source: BMC Med. 2023 Aug 4;21:289. doi: 10.1186/s12916-023-03005-3 (PMC10403852; doi:10.1186/s12916-023-03005-3)
Supplement: Supplementary file 3 — Additional file 3: Supplement Figure S1. Prevalence of social, physical, and psychological health characteristics in AYA survivors by race/ethnicity. The weighted proportion of AYA survivors by race/ethnicity group in our study. The greatest values were highlighted with red circle. ** Cardiovascular comorbidities: hypertension, stroke, angina/angina pectoris, myocardial infarction, obesity, diabetes mellitus, dyslipidemia; Non-cardiovascular comorbidities: arthritis, thyroid disease, asthma; Daily limitation: daily activity limitation due to emotional problem. [file 12916_2023_3005_MOESM3_ESM.pdf]

	NHW	AA	Hispanic	Asian
Poor general health (%)	18.9	12.9	24.9	32.8
**Social health**				
Low education	10.6	11.8	32.1	15.7
Non-couple	30.8	63.5	37.9	22.6
Low household income	11.9	26.6	29.6	14.3
Unemployed	41.1	46.4	28.8	42.5
**Physical health**				
Former/current smoker	53.4	47.5	23.8	23.5
Former/current drinker	94.6	95.0	78.4	89.2
CVD comorbidities**	36.2	51.9	19.2	17.5
Non-CVD comorbidities**	72.6	78.6	68.2	49.4
Pregnant, yes	85.6	94.4	91.1	89.6
Birth, yes	84.0	91.6	90.4	88.7
**Psychological health**				
Daily activity limitation**	5.0	2.8	5.1	3.8
Depression	3.0	2.8	1.2	9.2
Suicide ideation	4.7	3.0	5.4	10.7
